# Mapping lifestyle medicine in undergraduate medical education: a lever for enhancing the curriculum

**DOI:** 10.1186/s12909-022-03929-z

**Published:** 2022-12-20

**Authors:** Jumanah Essa-Hadad, Mary CJ Rudolf, Noah Mani, Lilach Malatskey

**Affiliations:** Department of Population Health, Azrieli Faculty of Medicine, Bar Ilan University, POB 1589, Henrietta Szold 8, 1311502 Safed, Israel

**Keywords:** Lifestyle medicine, Lifestyle curriculum, Lifestyle medicine teaching, curriculum mapping, medical education

## Abstract

**Background:**

In 2017, the Bipartisan Policy Center called for inclusion of lifestyle medicine (LM) in undergraduate medical education. Recognizing the requirement that lifestyle medicine should be an integral and integrated part of the curriculum, we undertook an in-depth mapping process to determine the extent of LM teaching at our Faculty, staff perceptions and the impact on medical students.

**Methods:**

The study utilized mixed methodology. In Phase 1 (Mapping) structured analysis of course syllabi were conducted followed by observation of teaching sessions throughout the pre-clinical and clinical years, recording content, the degree of coverage, and time allocated to LM Medicine. In Phase 2 (Impact and perceptions), students’ attitudes and confidence in LM counselling were ascertained by questionnaire (scale 1–4) on completion of second and fourth year of studies. Interviews were conducted with course coordinators.

**Results:**

Phase 1: Students received 58 hours of LM teaching, 49 hours pre-clinical and 9 clinical; 42 hours were dedicated to theoretical knowledge and 16 hours to teaching practical skills related to lifestyle behavior change. Nutrition received the most attention (18 hours), alcohol, sleep, smoking and sexuality the least. On completion of the internal medicine rotation, students (*n* = 48) agreed that LM guidance should be part of the physician’s role and that patients expected their physicians to be role models (mean ± sd; 3.4 ± 0.7). Students were fairly confident about providing general LM counselling (3.3 ± 1.1); but less so for exercise (3.0 ± 1.2), nutrition (2.7 ± 1.1), stress (2.5 ± 1.0), sleep (2.2 ± 1.2), and sexuality (2.1 ± 1.2). Staff recognized the importance of LM but reported time limitations and the need to bring in external experts to teach LM as challenges.

**Conclusions:**

Real-time mapping of teaching is a valuable way to ascertain teaching in practice. Based on our mapping process, redesign of curricula is needed to integrate more competency-based, experiential teaching, particularly in the clinical years.

**Supplementary Information:**

The online version contains supplementary material available at 10.1186/s12909-022-03929-z.

## Background

The World Health Organization (WHO) estimates that almost two-thirds of all deaths worldwide are due to non-communicable diseases, which are often the result of unhealthy lifestyle choices [1]. Guidelines call uniformly for lifestyle change as the first line of therapy for prevention and treatment of chronic diseases [2, 3]. Doctors in particular are recognized as having a special role in encouraging patients to adopt a healthy lifestyle [2–4], and analyses of physician behavior has shown that physicians who have healthy personal habits are more likely to encourage patients to adopt such habits [5].

Despite this well-recognized link between lifestyle and chronic disease, physicians often fail to provide guidance about healthy behaviours [6, 7], even though they believe it is their responsibility to do so [8, 9]. In one study in the USA [7], fewer than 50% of primary care physicians routinely provided patients with specific guidance on nutrition, exercise, or weight control. Physicians reported that barriers to providing counseling on lifestyle behavioral change include insufficient confidence, lack of knowledge and skills, and lack of time, compensation and resources [7, 10, 11].

Lifestyle medicine is emerging as a specialty that is gaining formal recognition around the world [12]. It incorporates the use of evidence-based lifestyle therapy interventions, including physical activity, nutrition, sleep, stress management, and positive social connections as a primary means to prevent, treat, and reverse chronic diseases [13]. In 2017, the American Medical Association (AMA) passed a resolution supporting the incorporation of lifestyle medicine curricula in medical schools [14]. However, despite recognition of its importance by the AMA and the American College of Lifestyle Medicine (ACLM), there remains inadequate lifestyle medicine training across the medical education continuum [15]. Multiple reports from physicians, residents, and medical students show the gaping void in LM training and education [10]. In 1985, the National Academy of Sciences recommended that 25-hours should be dedicated to nutrition [16] yet by 2010, only 27% of US medical schools had incorporated nutrition courses into their curricula [17] and very minimal physical activity promotion is taught [18]. The need to include lifestyle medicine teaching in undergraduate medical education is evident, yet remains a neglected area in most medical schools [19].

The Bar Ilan University Azrieli Faculty of Medicine was established in 2011 in the Northern periphery of Israel. Since its inception, we have been working on two parallel components: training our future doctors to develop LM competence and creating a supportive environment that promotes health and well-being of our students (the Healthy Faculty Initiative). Our aspiration is that in the future our medical students will have the knowledge, attitudes and competencies to provide LM guidance to their patients and serve as positive role models [20].

As part of this aim, we introduced a spiral lifestyle medicine curriculum between 2012 and 2016 which was integrated into three preclinical and one brief clinical course (see Table 1 below). The intention was to provide a basis for further teaching, particularly in the clinical years. We appreciated that there were likely to be constraints in introducing additional subject matter into medical studies, but hoped that further teaching and experience would take root in other courses.

In 2018 we determined to conduct a mapping process of the entire curriculum in order to: (a) determine the extent of LM teaching that was actually taking place within the curriculum and identify gaps; (b) determine the impact on medical students’ knowledge, skills and attitudes toward LM counselling; and (c) assess staff views and attitudes towards incorporating LM within their courses and clinical rotations.

## Methods

### Setting and population

The Bar Ilan University Azrieli Faculty of Medicine opened in 2011. It is located in the city of Safed in the Galilee, a region of significant social disadvantage and cultural diversity. The principal MD track is a 4-year program, the first 19 months consisting of pre-clinical training on the medical school campus, followed by two and a half years of clinical training in five hospitals in northern Israel. At the time of the study, 70 students were enrolled in the program per year. The average age of students was 28 years and 60% (*n* = 42) were female. This is a graduate level program, all students had at least a first degree.

### Core LM curriculum

The spiral lifestyle curriculum in its current form is a required course for all 4-year students (see Table 1). It aims to provide students with the lifestyle medicine competencies set by the ACLM, the American College of Preventive Medicine (ACPM) [21], and the Israeli Society of Lifestyle Medicine [22] and was adapted to meet the unique needs of our region. Learning involves lectures, as well as interactive and experience-based teaching methods, such as role play, counselling and coaching of a patient in the internal medicine rotation.

**Table 1:** BIU faculty of medicine’s required lifestyle medicine spiral curriculum

Medical year	Course	Topics Taught	Learning objectives	Teaching Methods	Teaching Hours
**First year**	Population Health	-Introduction to LM -Importance of physical activity -Basic introduction to healthy eating -Stress -Workshops on healthy cooking, yoga and Tabata exercise	Gain in knowledge on the importance of lifestyle medicine, acquisition of tools and skills to improve their own dietary habits, and exposure to different types of physical activity and stress reduction methods	Frontal Lectures Experiential learning	8
	Bio-energetics	-Tool for health behavior change and exercise prescription -Physical activity -Smoking -Nutrition and obesity -Sleep	Gain in knowledge about: the relationship between physical activity, smoking, nutrition, sleep and diseases; recommendations for physical activity, nutrition, sleep, smoking and treatment of obesity, acquisition of tools for supporting patients with behavior change.	Frontal lectures Small group discussions	16
	MAHAR-Social Responsibility in Medicine	-Mindfulness session -Exercise sessions	Exposure to mindfulness and yoga as tools to cope with stress and the opportunity to engage in different physical activity sessions.	Experiential learning	3
**Second year**	HILA- Clinical Skills	-Motivational interviewing -Theoretical lifestyle counselling -Nutrition	Gain in understanding and acquisition of skills of motivational interviewing to improve outcomes of health behavior change counselling	Frontal lectures Small group discussions Role play with actors Case based learning	10
**Third (clinical)** **year**	Preventive Medicine	-Importance of lifestyle behavior on chronic disease prevention/ treatment -Practice motivational interviewing for lifestyle behavior change with patients -Online course- practicing motivational interviewing [1]	Gain in knowledge on the importance of lifestyle behavior to prevent and treat chronic diseases; practice with patient health behavior change tools.	Frontal Lecture Case-based learning Small group discussions	7
**Total Hours**					**44**

### Phase 1: mapping the teaching of lifestyle medicine

An in-depth observational study was conducted to map the entire medical curriculum through a lifestyle medicine lens. The purpose was to assess what and to what extent lifestyle medicine related issues were taught. It was carried out in the framework of a final research project by two medical students (NM and IT). They conducted a structured analysis of all course syllabi using systematic documentation and extracted data on teaching sessions that related to LM, proposed timing and learning objectives. They then attended all lectures during the pre-clinical years and utilized a purposely designed tool (see Appendix 1) to allow them to systematically record content, the degree of coverage, and time allocated to nine LM topics (General lifestyle medicine; nutrition; exercise; stress; smoking; alcohol; sleep; sexuality and health; tools for behavioral change). During the clinical years, documentation included the 4 weeks of introductory lectures, ward rounds (IT) and tutorials in the various rotations.

The documentation was analyzed quantitatively allowing for calculation of hours dedicated to teaching the nine LM topics during both the preclinical and clinical courses, and whether the focus was on theoretical knowledge or practical skills.

### Phase 2: impact and perceptions

- Impact on students’ attitudes and self-confidence

We ascertained students’ attitudes, competence and confidence relating to lifestyle medicine and their views on the role of the physician, through an on-line questionnaire [23] [as described in a previous study [24]]. It was derived from an instrument that was designed and validated in Israel [23] and was administered to medical students at the end of their 2nd year (after the internal medicine rotation) and fourth year of studies as part of student feedback/evaluation of lifestyle medicine teaching.

- Nine items (detailed in table 3) were used to assess students’ self-perceived competence and skills in health behavior counselling (ranked 1-not at all, 5–very much) [23].
- Attitudes towards the physician’s role were ascertained using 2 items to assess physicians’ attitudes to lifestyle counselling (ranked 1-strongly disagree, 4-strongly agree). Students were asked to rate their agreement with two statements: a) ‘patients expect their doctors to be a role model for a healthy lifestyle’; and b) ‘giving advice regarding healthy lifestyle is the role of allied health care professionals, such as dietitians, but not the doctor’.
- Two open ended questions were also included to students’ views on gaps in the teaching of lifestyle medicine and recommendations on how to improve their competence in counselling patients about lifestyle.

Data analysis: Quantitative data were analyzed using descriptive analysis, means and standard deviations were calculated.

b. Perceptions of course coordinators and department heads

Semi-structured phone interviews were conducted with coordinators of the pre-clinical courses and Department heads of the main clinical rotations. Informed consent was obtained from all participants. They were asked their views regarding the importance of teaching lifestyle medicine subjects in general and specifically as part of their courses. Their attitudes towards incorporating further teaching of lifestyle issues in their courses were sought along with suggested changes, and any views they had on how lifestyle medicine teaching might be promoted. A semi-structured interview guide was developed and used to guide the interview. Detailed notes and full comments from the coordinators were written down during the interviews and common themes were then identified by the medical student (NM) with her supervisor (LM). Qualitative data were analyzed using thematic analysis to complement findings of the quantitative data.

## Results

### Phase 1: mapping of the curriculum

**Fig. 1:**
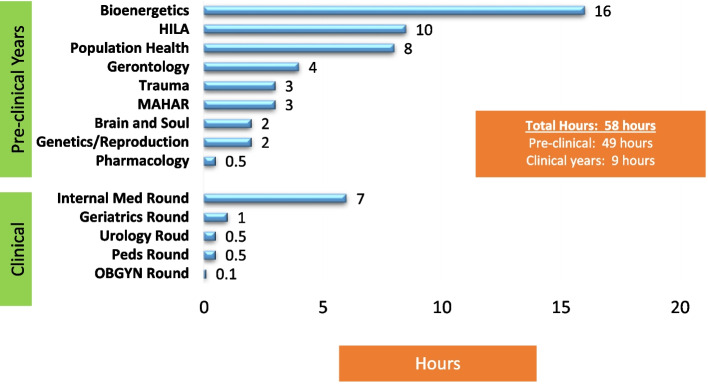
Lifestyle Medicine Hours Taught Across the Curriculum

An additional 14 hours of teaching were identified as related to lifestyle medicine beyond the 44 hours in the existing lifestyle medicine curriculum, giving students 58 academic hours of lifestyle medicine teaching across the entire four-year curriculum. Of the seventeen courses in the pre-clinical years, lifestyle medicine issues were taught in nine. In the clinical years, the students have five major rotations, namely internal medicine, surgery, pediatrics, psychiatry and obstetrics/gynecology, and several smaller rotations. Here, lifestyle medicine teaching was markedly less prominent with students having only 9 hours over 28 months (see Fig. 1). Of the total 58 teaching hours, the majority of time (42 hours) was dedicated to theoretical knowledge and 16 hours to teaching practical skills related to the encouragement of lifestyle behavior change (8 hours in HILA Clinical Skills course, 3 hours in population health, 2 hours in bioenergetics, and 3 hours in internal medicine).

**Fig. 2:**
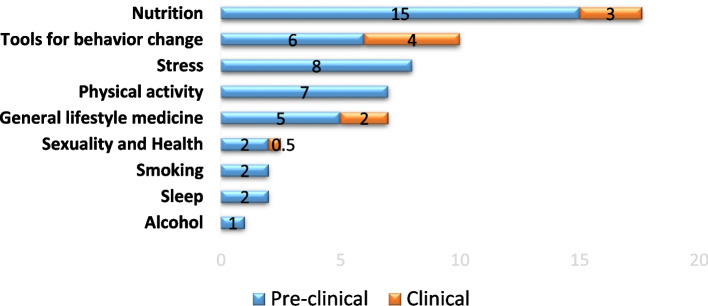
Lifestyle Medicine Topics Taught Across Curriculum

Figure 2 shows the results according to LM topics. Nutrition received the most attention (18 hours), followed by tools for behavior change (10 hours), stress (8 hours), physical activity (7 hours), and general lifestyle medicine (7 hours). Alcohol (1 hour), sleep (2 hours), smoking (2 hours), and sexuality and health 2.5 hours) were the issues taught least.

### Phase 2: impact and perceptions

#### Students’ attitudes to lifestyle medicine

48 of 70 (68%) students completed the questionnaire at the end of the 2nd year (after the internal medicine rotation) and 8 of 61 (13%) completed the questionnaire at the end of studies in year 4. As the response rate was so low at the end of studies, these results were not analyzed. Table 2 shows the students’ attitudes towards lifestyle medicine at the end of the second year. There was general agreement with the statement that patients expected their physicians to be role models for healthy lifestyle (mean 3.4, SD 0.7) and disagreement that giving advice regarding healthy lifestyle is the role of allied health care professionals (such as a dietitian) and not of the doctor (mean 1.7, SD 0.9).

**Table 2:** Attitudes towards lifestyle medicine and the physicians’ role (1 = Strongly Disagree; 4 = Strongly Agree)

Variable	n	Mean	SD
Patients expect their doctors to be role models for a healthy lifestyle	47	3.4	0.7
Advice regarding healthy lifestyle is the role of allied health professionals and not the doctor	48	1.7	0.9

### Students’ self-perceived confidence to provide lifestyle medicine counseling at the end of their second year (following the internal medicine rotation)

Table 3 shows students’ self-perceived confidence in providing lifestyle counseling to patients at the end of the second year. Although students reported some confidence in providing general lifestyle medicine advice, confidence levels were low when it came to providing guidance in specific lifestyle areas.

**Table 3:**
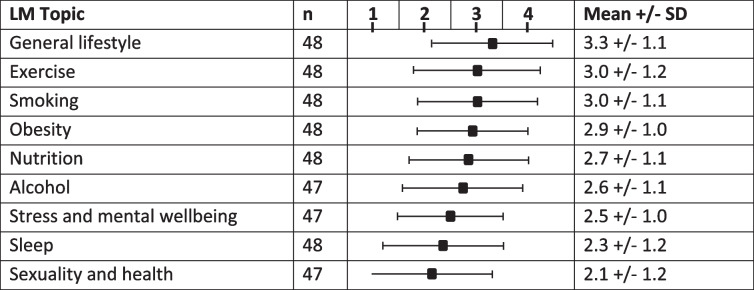
Self-perceived confidence in providing LM counseling (1–4; 1 = very low confidence; 4 = very high confidence)

Confidence was particularly low (mean +/− SD) in providing advice on coping with stress (2.5 +/− 1.0), sleep (2.2 +/− 1.2), and sexuality and health (2.1+/− 1.2). Despite nutrition being the LM topic taught most in the medical curriculum, self-perceived confidence in providing nutrition counselling was relatively low (2.7 +/− 1.1).

In the open-ended questions, students indicated several topics that needed more focus in the curriculum, including nutrition, practical tools for behavior change, such as motivational interviewing, offering physical activity sessions and general lifestyle promoting activities. Their recommendations regarding what further was needed to improve their ability to provide counselling included adding more exposure to LM information and skills, offering additional opportunities to practice LM counselling with simulations and role play, and providing more opportunities for students to practice healthy lifestyle behaviors themselves. The following quote from one student reflects a reoccurring theme for a much stronger focus on LM teaching:

*“I wonder if lifestyle medicine is the focus of attention of our health care system? It seems that lifestyle medicine can be addressed in every clinical round, however there does not seem to be systemic backing. I have never encountered a ward or hospital director who talks about the importance of sports, proper nutrition, and maintaining a healthy mind as a prevention of chronic diseases... This is not right!”* (4th year male medical student).

### Perceptions of course coordinators and department heads

Semi-structured interviews were conducted with 15 of 21 coordinators of pre-clinical courses and five Faculty leads for the principal departments providing clinical rotations. In general, the interviewees recognized the importance of lifestyle medicine. As one pre-clinical coordinator stated:

*“A healthy lifestyle is an important issue and should be devoted to a course in itself. It is one of the most neglected areas for doctors.”* (Pre-clinical trauma course coordinator).

This view was also evident among clinical year department heads:

*“I think in clinical rotations more emphasis should be placed on the issue of preventive medicine. In each team meeting we should devote a quarter of an hour / half an hour to these issues, almost every day. If they were to extend the rotation, I would be happy to involve good experts who know how to provide counselling including some from epidemiology and preventive medicine.”*

(Faculty Head for Internal Medicine)

Time was seen to be the major constraint particularly in the clinical rotations, where the issue of time was raised by all – both in relation to provision of guidance for patients as well as teaching for students. To quote the Faculty Head for Internal Medicine:

*“In nutrition and all other lifestyle issues you need to find time to combine them within the rotation and with the teaching. It is essential and very important I think. The problem is that the rotation is short and it has to be extended, to meet all these issues.” (*Faculty Head for Internal Medicine.)

Some Heads of Department felt that lifestyle medicine would be better taught in the community or primary care rather than in hospital. One interviewee was clear that her Department was not the setting to address lifestyle medicine:

*“We do not dedicate time to the topic in this rotation, except for specific mentions such as: exercise - in the context of uterine cancer prevention. Nutrition – during pregnancy. Sexuality - in the context of post-surgery and radiation. And usually correlated to all sorts of pathologies.”*

(Head of Department of Gynecology and Obstetrics)

## Discussion

Recognizing the requirement that lifestyle medicine should be an integral and integrated part of the medical education curriculum, we conducted an in-depth mapping process with the aim of determining the extent of LM teaching at our Faculty, along with the impact on medical students’ attitudes and confidence toward LM counselling, and staff perceptions on the importance of LM teaching.

We learned that in practice, students were provided with 58 hours of LM teaching across the four-year program, with very little taking place in the clinical years. It was disappointing to discover that the teaching was principally located in our core spiral lifestyle medicine curriculum, and did not significantly extend across other courses. In comparison to ‘gold standard’ LM curricula, such as the University of South Carolina Greenville Medical School, which provides 86.5 hours of LM teaching in their curriculum [10], we have much room for improvement. While nutrition is the LM topic taught most in our curriculum, at 18 hours we still do not meet the National Academy of Sciences recommendation of 25 hours of nutrition education in medical schools [16]. Our curriculum only includes 7 hours of physical activity teaching, which is less than the average training of 8 hours in American medical schools [25]. This is concerning, since physical inactivity and sedentary behavior are key modifiable risk factors for the development of chronic diseases [26]. Other areas that need significant improvement in our curriculum are smoking, stress, sexuality, and alcohol.

All course coordinators in the pre-clinical years and clinical heads of departments recognized the importance of lifestyle medicine. However, although they were supportive, they reported time limitations as a major challenge to teaching LM. Some also indicated the need to bring in external experts to teach lifestyle medicine. They only commented on LM being preventative rather than curative treatment. Furthermore, they saw the community as the setting where LM should be taught.

Students also recognized the importance of lifestyle medicine and by the time they had completed their internal medicine rotation, they had positive attitudes regarding the importance of the physician’s own lifestyle and the responsibility of doctors to provide lifestyle counseling rather than leave it to para-professionals. However, self-perceived confidence levels in providing lifestyle medicine counseling were low.

As the leads for lifestyle medicine in the Faculty, we invested much time in planning and implementing lifestyle medicine teaching. We were disappointed to see that self-perceived confidence levels were not higher, likely to be due to inadequate opportunities to practice lifestyle guidance in the clinical years and the lack of role-modelling by medical staff. More opportunities are needed through experiential learning, such as simulations and role play during the pre-clinical years and practice with patients during the clinical years.

### Strengths and limitations

Our research is novel as we mapped the entire curriculum in our medical school through observation of teaching, rather than simply through survey of course curricula and syllabi. This was conducted in real time by medical students sitting in on the lectures and teaching sessions over a period of 2 years utilizing a specifically designed tool for the purpose. It was an arduous exercise but of real value in understanding the field in practice, and allowed us to ascertain what is taught in practice rather than relying on syllabi which may reflect intentions to teach rather than reality. It also exposed the lack of competency teaching, at a time when competency-based medical education is at the forefront, with lifestyle medicine and skills being an explicit requirement. For example, the General Medical Council in the UK demands that doctors in training must ‘demonstrate basic principles of public health, including promoting health and wellbeing, nutrition, exercise and illness prevention [27].

There are limitations to our study that need to be considered. Firstly, our mapping exercise was largely quantitative, although students’ comments allowed a sense of the depth and quality of the teaching too. Also, due to limited resources, most of the mapping during the clinical years was carried out in the affiliated hospital closest to the medical faculty campus. It is unlikely that teaching differed significantly in the other affiliated teaching hospitals since they all follow the same syllabus. Nonetheless, caution is required before generalizing across the whole of clinical studies in our medical school. Unfortunately, it was hard to ascertain the extent of LM teaching in the Family Medicine rotations as students are principally taught individually in primary care clinics, which are likely to vary greatly in their focus on lifestyle medicine. Another limitation of the study is that we checked LM knowledge and confidence, but did not have a record of competence from observation or OSCE exams. Lastly, we received a poor response to questionnaires at the end of medical training so we could not ascertain students’ ultimate confidence and attitudes. It is hard to believe that, given the lack of teaching beyond the internal medicine rotation, it would be likely to increase.

### Implications for practice

Our study has led us to provide some recommendations in practice (see Table 4). With the gap in LM teaching between the pre-clinical and clinical years, our next steps must focus on finding ways to ensure that time for LM teaching is allocated in the clinical years. An emphasis must be placed additionally on incorporating LM teaching in areas that are lacking, such as sexuality, sleep, alcohol, and stress. The adage that ‘assessment drives learning’ is particularly apt and lifestyle medicine will only get its justified place in the curriculum if students are assessed on their competence in this area. While it would be relatively simple to introduce LM into written assessments, the real issue is to assess abilities in counselling patients around behavior change. There is however a notable lack of role models in teaching and demonstrating LM clinical skills in routine patient care. Faculty development is therefore required and a focus is needed on using lifestyle medicine counselling as a routine part of teaching in the hospitals. Faculty heads, not surprisingly, stated that time limitations were the biggest constraint. This can only be overcome by strategic planning at the administrative level with greater emphasis placed on LM.

**Table 4:** Future recommendations for moving forward

	Recommendation
**1**	Faculty development on teaching lifestyle medicine competencies.
**2**	Increase teaching on all areas of lifestyle medicine, according to international guidelines.
**3**	Ensure that time for lifestyle medicine teaching is allocated in both the pre-clinical and clinical years.
**4**	Incorporate experiential learning to ensure that students gain competence in lifestyle medicine.
**5**	Mapping as an important adjunct to ensure that lifestyle medicine is an integral and integrated part of the medical education curriculum.
**6**	Introduce lifestyle medicine assessment in OSCE pre-clinically and clinically.

### Implications for policy

This research is of relevance to medical educators and also at the health policy level. In 2010, the AMA declared its support for legislation that incentivizes and provides funding for the inclusion of lifestyle medicine education in medical school education [27]. More recently, a symposium of leading health organizations, was convened by the Bipartisan Policy Center in the USA, calling for the inclusion of nutrition and physical activity at all levels of medical education (https://bipartisanpolicy.org/download/?file=/wp-content/uploads/2019/03/BPC-Training-Health-Professionals-for-Obesity-Care.pdf). Other action has included a demand for state and federal support for impactful and lasting change within the delivery of medical care, with the initiation of a think tank with the remit of opening communication, informing local- and national elected officials, and addressing potential necessary policy challenges (https://www.acpm.org/getmedia/1991b553-f955-494c-a795-1d31d587aa5f/lifestyle_medicine_legislati.pdf.aspx) [28]. As our study highlights, 20 years on, these requirements are far from being adequately implemented.

## Conclusion

Real-time mapping of teaching in the medical curriculum is a valuable way to ascertain how LM is taught in practice. Based on our mapping process, it is necessary to redesign our curriculum to integrate more competency based, experiential LM teaching across the general medical education curriculum, particularly in the clinical years. Medical schools throughout the world can learn from our experience and challenges and more effectively develop and adapt their curriculum to incorporate LM teaching for the next generation of doctors.

## Supplementary Information

**Additional file** **1.**

## Data Availability

Datasets and other materials are available upon request from corresponding author.

## Abbreviations

WHO: World Health Organization
LM: Lifestyle Medicine
AMA: American Medical Association
ACLM: American College of Lifestyle Medicine
ACPM: American College of Preventive Medicine
OSCE: Objective Structured Clinical Examination
