# Supplementary material for: Mapping lifestyle medicine in undergraduate medical education: a lever for enhancing the curriculum
Source: BMC Med Educ. 2022 Dec 20;22:886. doi: 10.1186/s12909-022-03929-z (PMC9769064; doi:10.1186/s12909-022-03929-z)
Supplement: Supplementary file 1 — Additional file 1. [file 12909_2022_3929_MOESM1_ESM.pdf]

Appendix I: Example of Lifestyle Medicine Teaching Documentation

Name_____________________________________ Date____________________ Year____________

Topic: General lifestyle medicine

Name of course: ____

Lesson: ____

Total duration: ____

Main topics	How topic is taught: Lecture/Small Group Sessions/Simulations/Case study/Other	Quality of LM teaching (1-5)*	Time spent on topic	Comments
Lifestyle medicine, the importance of treatment using healthy lifestyle				
The relationship between lifestyle, health and chronic diseases				
The importance of the doctor's health as a role model				
Experiential and practical experience related to lifestyle components, such as developing a personal lifestyle plan				

*Scale: 1- Very limited; 5- Highly relevant

Additional comments and recommendations for the future (For example, if there were opportunities to teach additional lifestyle medicine topics:

____

____
